# An Overview of Current Knowledge on *in vitro Babesia* Cultivation for Production of Live Attenuated Vaccines for Bovine Babesiosis in Mexico

**DOI:** 10.3389/fvets.2020.00364

**Published:** 2020-06-26

**Authors:** J. Antonio Alvarez, Carmen Rojas, Julio V. Figueroa

**Affiliations:** Laboratory of Bovine Babesiosis, National Institute for Forestry, Agriculture and Livestock Research (INIFAP), National Disciplinary Research Center on Animal Health and Safety (CENID-SAI), Jiutepec, Mexico

**Keywords:** *Babesia bovis*, *Babesia bigemina*, *in vitro* cultivation, production, live attenuated vaccines

## Abstract

The instrumentation of the *in vitro* culture system has allowed researchers to learn more about the metabolic and growth behavior of *Babesia* spp. The various applications for *in vitro* cultivation of *Babesia* include obtaining attenuated strains for vaccination or pre-munition, the selection of pure lines with different degrees of virulence, studies on biological cloning, ultrastructure, antigen production for diagnostics, drug sensitivity assessments, and different aspects of parasite biology. Although there are different types of vaccines that have been tested against bovine babesiosis, so far, the only procedure that has offered favorable results in terms of protection and safety has been the use of live attenuated vaccines. In countries, such as Australia, Argentina, Brazil, Uruguay and Israel, this type of vaccine has been produced and used. The alternative to live vaccines other than splenectomized calf-derived biological material, has been the *in vitro* cultivation of *Babesia bovis* and *B. bigemina*. The development of *in vitro* culture of *Babesia* spp. strains in a defined medium has been the basis for the initiation of a source of parasites and exoantigens for a variety of studies on the biochemistry and immunology of babesiosis. The use of live immunogens from attenuated strains derived from *in vitro* culture is highlighted, which has been proposed as an alternative to control bovine babesiosis. In several studies performed in Mexico, this type of immunogen applied to susceptible cattle has shown the induction of protection against the experimental heterologous strain challenge with both, *Babesia*-infected blood and animal exposure to confrontations on tick vector-infested farms. The combination of transfection technologies and the *in vitro* culture system as integrated methodologies would eventually give rise to the generation of genetically modified live vaccines. However, a greater challenge faced now by researchers is the large-scale cultivation of *Babesia* parasites for mass production and vaccine distribution.

## Introduction

Bovine babesiosis, also known as Texas fever, “tristeza,” tick fever or red water, is caused by intraerythrocytic protozoa of the genus *Babesia* that are transmitted by ticks. They can produce an acute disease with clinical findings characterized by fever, hemolytic anemia, hemoglobinuria and death; but abortion can be caused in pregnant females after the first third of pregnancy (1, 2). Clinical signs vary depending on the pathogenicity and virulence of the species and strain of *Babesia*. There are factors that determine the bovine babesiosis infection, such as age, race and immune status of the animal. Affected animals, recovering from the acute disease, usually remain as asymptomatic carriers for years, can become reservoirs of infection for healthy animals and hardly reach the production levels that were lost by the chronic infection (3).

In Mexico, *Babesia* parasites are transmitted mainly by *Rhipicephalus microplus* ticks and the species so far identified are *Babesia bovis* and *B. bigemina* (4). Depending on the predominant species, there are variations in the pathogenesis and course of the disease (4, 5). In cattle infected with *B. bovis*, the presentation is severe and is characterized by high fever, ataxia, anorexia and general circulatory shock. Nervous signs are regularly associated with the sequestration of infected erythrocytes in the cerebral capillaries. Infection with *B. bigemina* usually manifests more benignly, but infected cattle may present with more severe hemolytic anemia (6). Babesiosis is currently considered as one of the main obstacles to the development of livestock in tropical and subtropical regions of the world. It directly affects the production of meat and milk, affecting the competitiveness of livestock industries (5, 7).

For the control of the disease there are different strategies such as the use of ixodicides for vector control, controlled translocation of cattle, chemotherapy, chemoprophylaxis and selection of tick-resistant cattle. These procedures are effective only if they are included in an integrated control program, which can be costly and impractical (8). Immunization of cattle is currently considered to be the most appropriate procedure for prevention and control of bovine babesiosis; This has been demonstrated with favorable results in terms of protection and safety (9).

## Geographic Distribution

There are more than 70 species of protozoa of the genus *Babesia*, distributed around the world, 18 of which can cause disease in different domestic mammals (10). Four species stand out due to their economic importance in affecting cattle: *B. bovis* (11), *B. bigemina* (12), *B. divergens* (13), and *B. major* (14). The most important, from an economical point of view, are *B. bigemina* and *B. bovis*, widely distributed in areas where their arthropod vectors exist, which are ticks such as *R. microplus, R. decoloratus, R. annulatus*, and *R. geigyi*. *B. bigemina* can also be transmitted by *R. decoloratus* and *R. evertsi* (1, 15). *B. divergens* is transmitted by *Ixodes ricinus* (16), and *B. major*, transmitted by *Haemaphysalis punctata* (16). *Babesia* is distributed in countries located between 30°S and 40°N of the equator an area that corresponds to the presence of its arthropod vector, *R. microplus, R. annulatus*, and *R. decoloratus* (16).

## Economic Importance of Babesiosis

Bovine babesiosis is a serious problem for livestock, especially in developing countries, as they limit the introduction of European type livestock specialized in meat and milk production to tropical regions. In Mexico, bovine babesiosis, described for the first time in early 19th century (17) continues to be a limiting factor for cattle industry production, as the tick vector is distributed in the main tropical livestock production regions. The economic importance of the disease is reflected by the high morbidity and mortality rates in livestock (18).

Due to its wide distribution and effects on livestock (1) bovine babesiosis has been considered the most important among arthropod-borne diseases in cattle (5). Numerous economic losses due to babesiosis, anaplasmosis and ticks have been estimated in different countries of the world. Economical annual loss in amounts of $ 23.3, $ 5.1, $ 5.4, $ 6.8, $ 21.6, $ 19.4, $ 57.2, $ 3.1, and $ 0.6 million USD have been calculated for Australia, Kenya, Zimbabwe, Tanzania, South Africa, China, India, Indonesia and the Philippines, respectively (5). More recently in Mexico, the potential economic losses related to milk and beef production and associated exclusively to the cattle tick *R. microplus* were estimated at $ 573.61 million USD per year (8). Although well recognized by authorities that there is high mortality as well as drop in milk, meat and calves production caused by babesiosis, a current estimate of the real economic impact of the disease in cattle production is not yet available (8). It has been well established that the *Babesia* tick complex hinders the importation or mobilization of genetically superior cattle to tropical regions (16, 19). The economic impact of babesiosis with morbidity rates above 50% in endemic areas, reflects the high risk for occurrence of outbreaks in susceptible animals in the Mexican tropics (4).

## *Babesia* Replication in the Host Cell

The first step in invasion is the apparent random interaction between the merozoite and the host cell. For cell invasion to occur, there must be recognition between the surface components of the parasite membrane and the host erythrocyte that leads to the molecular coupling of the surfaces of both cells (20). The erythrocyte invasion by *Babesia* merozoites is summoned in five steps: (a) Contact between merozoite and erythrocyte; (b) Orientation of the apical pole of the merozoite to the surface of the erythrocyte; (c) Fusion between merozoite and erythrocyte membranes; (d) Release of the rhoptry contents; (e) Erythrocyte membrane invagination. The invasion process occurs when the sporozoites and subsequently the merozoites encounter the erythrocytes. Then, the sporozoites place their apical portion against the surface of the erythrocytes and secrete histidine-rich proteins, contained in the rhoptries and micronemes. Proteins secreted on contact with the membrane facilitate the entry of the parasite into the erythrocyte, without an exoerythrocytic phase. A parasitophorous vacuole is then formed which, by electron microscopic studies on *B. microti* in hamster erythrocytes (21), and on *B. divergens* in human erythrocytes (22) was demonstrated to be lost soon upon invasion, leaving the parasites in direct contact with the cytoplasm of the host cell. The parasite is not isolated from the external environment though, suggesting that intraerythrocytic *Babesia* uses a novel trafficking mechanism for export of proteins into the host. It was shown recently, that vesiculation at the parasite plasma membrane of *B. microti* produces an interlacement of connected vesicles in the host erythrocyte responsible for the export of antigens from the parasite to the host erythrocyte and subsequently to mouse plasma (23). The parasite differentiates to form the trophozoites (rings), which are then divided by binary fission and give rise to two (or four in the case of *B. microti* and *B. divergens*) merozoites. These are united at an angle and once they mature, they separate, leave the erythrocyte and continue to invade new erythrocytes (24).

## Control of Bovine Babesiosis With the Use of Immunogens

It is frequently highlighted that in order to prevent and/or control bovine babesiosis in endemic countries, the primary goal has been to develop adequate immunogens using live or recombinant vaccines. Conventional procedures so far used and recommended for bovine babesiosis control are: vector control by using ixodicides; controlled mobilization of livestock to prevent asymptomatic and tick-infested livestock from being taken to free zones; both chemotherapy and chemoprophylaxis can be tactically included in a comprehensive program, although they are costly and impractical; the use of resistant livestock has been common in some countries with unsatisfactory results (25). Immunization is the procedure that has been identified for many years as the most appropriate way to prevent and control bovine babesiosis (9).

Historically, premunition has been used for the protection of cattle against babesiosis, which consists of the sub-inoculation of blood from an asymptomatic carrier bovine to susceptible cattle (26). Currently that form of prevention is not recommended because severe disease outbreaks can occur, and other pathogens can be transmitted. This procedure has been empirically applied, and may lead to the spread of other diseases - tuberculosis, brucellosis, IBR, etc. Premunized animals can be clinically affected with babesiosis and may even die if not treated in a timely manner.

Technically supported immunoprophylaxis procedures have been attempted for many years. In Australia, research began with the use of living organisms of reduced virulence, consisting of suspensions of *B. bovis* or *B. bigemina* infected erythrocytes with parasite populations previously attenuated by multiple passages in splenectomized or intact calves, respectively, from which blood is obtained with high parasitemias for application as a vaccine (27–31). Some limitations for this type of biological material are the need of an efficient cold chain maintenance and potential risk of contamination with other adventitious pathogens.

Antigens from erythrocytes and plasma from infected animals have also been used. These have provided some protection when applied with Freund's adjuvant or with saponin (32). At about the same time, the *in vitro* cultivation of *Babesia* was developed, a system from which attenuated strains that have functional immunogenic proprieties have been derived and tested. These strains have been used both in controlled and field studies, in which protection in vaccinated cattle of at least 80% has been demonstrated when challenged with virulent strains (33–36).

With the use of the *in vitro* culture system, parasite genes have been identified and cloned in vector expression systems to express recombinant proteins in *E. coli*, which have subsequently been produced, purified and used as immunogens, highlighting for the case of *B. bigemina* the GP45 protein which is a membrane surface glycoprotein (37); AMA1 integral membrane protein (38), and RAP-1 protein associated with rhoptries (39); whereas for the case of *B. bovis* the MSA-1 and MSA-2 proteins which are membrane surface glycoproteins and 12D3, a protein associated with the parasite surface and released into the erythrocyte stroma (15). It has been shown that these recombinant proteins generate high antibody titers in immunized cattle that are capable of inhibiting parasite invasion *in vitro*. However, *in vivo* trials demonstrated lack of protection against parasite challenge with field isolates (40, 41). Moreover, cattle immunized with a multi-epitope modified vaccinia Ankara virus and recombinant proteins were not protective after challenging by a virulent *B. bovis* strain (41).

Recent reviews on the application of molecular type technologies for development of immunogens against babesiosis have reported that effective immunogens against the disease has not been achieved yet. In contrast, it has been described that only live vaccines, whether derived from *in vitro* culture or from sub-inoculation in splenectomized calves, have demonstrated satisfactory protection in immunized animals against challenge with virulent parasites (15, 42, 43).

## History of *Babesia in vitro* Cultivation

The establishment of the *Plasmodium* culture was fundamental for *Babesia in vitro* cultivation. In this process, several culture factors were determined to be critical such as; good management sterile practice, use of defibrinated blood, removal of white cells from the cell pack, appropriate incubation temperature, glucose addition, anaerobic conditions and fluid levels (44). Continuous replication cycles were achieved in a velobiosis atmosphere by burning a candle inside a desiccator (45). The need to produce biological material for biochemical, immunological or chemotherapeutic studies of bovine babesiosis was of utmost interest for researchers that developed procedures for establishing the *in vitro* cultivation of *Babesia* spp.

For *Babesia* parasite culture assays, the target cells are mature erythrocytes, which provide the appropriate metabolic requirements for the growth and replication of the intraerythrocytic parasite stages (46). Erythrocytes from a carrier of the same species were shown to be a determining factor, as the proliferation of *B. microti* was achieved only in hamster erythrocytes by supplementing the culture medium with bovine fetal serum, rat serum or hamster serum (47).

In Mexico, similar procedures were applied to establish the short term culture of *B. bovis*, for which 50% erythrocyte were resuspended in culture medium 199 with Earle's salts supplemented with 50% bovine serum, in spinner flasks kept under constant agitation and incubation at 37°C in a 5% CO_2_ atmosphere in air (48). Such studies favored the initiation of *B. bovis* trials under similar laboratory conditions. The continuous growth of the parasite was established in Mexico for the first time worldwide. Bovine erythrocytes were kept in suspension in culture medium 199 added with HEPES (N-2 hydroxymethylpiperazine N1-2 ethanosulfonic acid) as buffer and supplemented with 50% bovine serum. The pH was adjusted to 7.0 maintaining an atmosphere of 5% of CO_2_ in air; the complete suspension was kept in agitation at 100 rpm and incubation at 37°C (48). For other *Babesia* species, such as *B. divergens*, maintenance in human cells was achieved for 3 days in a supplemented medium with 10% of bovine fetal serum, adding antibiotics, HEPES buffer salt, and incubating at 37°C (49).

Subsequently, by incorporating some modifications to the culture system, the continuous growth was maintained for 32 days, with percentages of parasitized erythrocytes (PPE) reaching 15%, showing that their virulence in cattle remained without changes (50). By studying additional variables, no difference was found when using RPMI 1640, 199, or NCTC-135 as culture media, but it was demonstrated that a pH <6.7 or >7.3 adversely affected the parasite's growth (51). The success so far obtained for *in vitro* growth of *B. bovis*, allowed an improvement in the methodology and the development of an *in vitro* cultivation system named Microaerophilic Stationary Phase (MASP) system, avoiding the cultivation of infected erythrocytes in suspension in spinner flasks. In this system, fluid levels in the culture medium were maintained in an atmosphere of 5% CO_2_ in air. The depth of the fluid level in the culture vessel was identified as a determining factor. Under these conditions the MASP system allowed an increased invasion of parasites to normal erythrocytes in cumulative dilution levels of up to 1.7 × 10^10^ over a period of 83 days (52). The development of *in vitro* cultures for *B. bigemina* and *B. rodhaini* was also attempted, with positive results but just for short periods (53).

Additional modifications for *in vitro* cultivation of *B. bovis* included the replacement of HEPES by TES (N-Tris hydroxymethyl methyl 2-aminoethanic acid) as buffer salt (52). This change in buffer also achieved the adaptation and conservation of different parasite strains and allowed that *B. bovis* could be recovered *in vitro* by implementing a cryopreservation procedure (54). Biological cloning by limiting dilution techniques was later developed and three *B. bovis* parasite lines were established *in vitro* (55). It is noteworthy to mention that in the case of *B. bovis*, erythrocyte infections exceptionally reach 2% of parasitized erythrocytes (PPE) in cattle, in contrast to the *in vitro* cultivation system in which PPEs can be much higher (up to 10%), while with Percoll gradient concentration procedures the PPE can reach at least 49% (56) and even higher for *B. bigemina* (57).

With the experiences generated for *B. bovis*, the continuous cultivation of *B. bigemina* was soon established, reaching PPE of 3–6% (58). Similar to what was accomplished with *B. bovis*, complementary methodologies for *in vitro* cultivation were developed for cryopreservation (59) and cloning of *B. bigemina* parasites (57). Thus, with the thoroughly described procedures, the cultivation of *B. bovis* (60) and *B. bigemina* was routinely established in Mexico (61).

Overall, the *Babesia in vitro* culture system is a procedure consisting of a suspension of erythrocytes from a bovine donor in a chemically “defined” culture medium, supplemented with adult bovine serum. Complete medium is the source of nutrients for the growth and multiplication of parasites. *In vitro* cultivation of *Babesia* parasites has allowed researchers to identify some nutritional needs of this parasite (62); to study different aspects of parasite biology and its life cycle (43, 63); host-parasite relationships and the identification of proteins involved in the invasion process (64). It has also facilitated to gain knowledge on the metabolic, physiological and reproductive behavior of these protozoa. Biochemical studies of parasite cell lines have been obtained after being biologically cloned (55, 65). In addition, the culture system has been used as a source of soluble antigens for immunoprophylaxis studies (66); to conduct research on parasite ultrastructure (67); for preparation of antigen for serological diagnosis (68, 69); and to conduct drug sensitivity studies (70, 71). Above all, the use of live immunogens derived from *in vitro* culture has been highlighted as an alternative in the control of bovine babesiosis (15, 31, 33, 34, 72). This topic will be further described in section “Importance and immunoprophylactic applications of *in vitro* culture-derived parasites.”

## Elimination of Bovine Serum in the Culture Medium and Supplementation With Vital Molecules for the *in vitro* Growth of Some Protozoa

The culture medium is the most important component of the culture environment, as it provides the necessary nutrients, growth factors and hormones, and it regulates the pH and the osmotic pressure. In initial cell culture experiments, natural media obtained from tissue extracts and body fluids were used. The need for standardization of media quality and increased demand led to the development of defined media classified as: (a) basal media; (b) media with reduced serum addition; and (c) serum-free media (73). Basal media contain amino acids, vitamins, inorganic salts and glucose as a carbon source, these formulations must be supplemented with serum. Formulations with low serum concentration need to be enriched with nutrients and animal derived factors. Serum-free media requires replacement with nutritional formulations containing growth factors and hormones; the latter are selective means for specific cell lines (74, 75). The propagation of cell cultures *in vitro* is used as a research model and is currently being used to eliminate the use of animals for experimental purposes (76). Innovations in cell culture have been continuously generated and increased, for application in immunology, pharmacology, physiology, toxicology and oncology among many other applications (77). However, in most studies, the culture depends on the required use of animal derived products, particularly fetal bovine serum as part of the culture medium, as an indispensable factor for cell growth and proliferation (78). Calf serum is a not well-defined supplement, because it varies quantitatively and qualitatively in its composition between batches. In addition, it may contain different amounts of endotoxins, hemoglobin or other adverse factors (79). It can also be a source of viral, bacterial, fungal or parasitic pathogens. Due to all these variables it has been suggested to avoid serum in cell culture *in vitro* (74, 80), to the extent that there are at least 450 different serum-free commercial media, which function only for a small number of cell types. It has even been indicated that if cell cultures could be available without the use of serum of animal origin, the standardization of protocols and replicas of the experiments between researcher groups could be established (74, 81).

The *in vitro* cultivation of *B. bovis* and *B. bigemina*, routinely requires the basal medium M-199 with Earle's salts and RPMI-1640, invariably supplemented with bovine serum in high proportions, from 20 to 40% (82, 83). Despite its importance, research on *in vitro* cultivation is carried out in just a few laboratories in the world and there are reports associated with the development of culture systems using chemically defined media. Growth factors to increase the PPEs, such as the replacement of adult bovine serum with fetal bovine serum, equine serum, serum fractions, bovine serum albumin or lipoproteins have been identified (82). For example, in *B. divergens* cultures, high density lipids (HDL) have been added and the development of cultures with high PPE has been demonstrated (84). In addition, high parasite proliferation with fetal bovine serum reaching a PPE similar to that obtained with adult bovine serum has been described (85). By using a medium enriched with ALBUMAX II, which is a complex mixture of lipids, and hypoxanthine as serum substitute it was possible to grow *B. bovis* (62). Exclusion of bovine serum for *in vitro* cultivation of *B. bovis* was possible by using the GIT medium (70).

Similarly, for the maintenance of *B. caballi*, the horse serum has been replaced by bovine serum albumin and chemically defined lipids (86). With the addition of hypoxanthine, the concentration of horse serum or fetal bovine serum could be reduced to 10% (87, 88). However, for the *in vitro* cultivation of *B. bigemina*, the concentration of bovine serum only was reduced to 20% and for short periods of time (89). The interest in eliminating the animal serum component has generated the commercial availability of different media for cell cultures, of which between 80 and 90% include albumin, transferrin and insulin in its composition (90). Chemically defined media have been developed in recent years, with formulations that partially or totally reduce bovine serum, without altering the morphology and function of cultivated cells. These new media containing vital elements for eukaryotic cell lines, had not been used until very recently for the *in vitro* cultivation of protozoan parasites where the maintenance and proliferation of *Babesia* spp. was accomplished (91, 92).

In Mexico, it was shown that *B. bovis* and *B. bigemina* can be adequately controlled for *in vitro* proliferation in a bovine serum-free medium with supplements such as insuline (ins), transferrine (trans) and selenite (sel) at optimal concentrations (91–93). The Advanced DMEM/F12 culture medium (A-DMEM/F12) supplemented with 40% adult bovine serum (v/v), was selected as an alternate medium to the traditional M-199 with Earle's sales (M-199). Improved parasite growth determined as maximal PPE reached, 9.59 and 8.37%, were obtained for *B. bovis* and *B. bigemina*, respectively, and the frequency for splitting cultures was reduced to every 24 h; This modification was possible because PPEs >4% were determined. Additionally, with the use of A-DMEM/F12, a period of adaptation of the parasites to the new culture medium was not necessary (91). The A-DMEM/F12 medium proposed in that study included the minimum concentration of the ins-trans-sel mixture as essential components to replace the bovine serum. It has been noted that the concentration of this mixture will depend on the needs of each cell culture (73, 79, 90). When A-DMEM/ F12 medium was supplemented with the ins-trans-sel mixture (at 2000, 1100, 1.34 mg/L, respectively), the proliferation of *B. bovis* (maximal PPE 9.73%) and *B. bigemina* (maximal PPE 7.23%) was constantly stimulated, occurring this without an adaptation period, and no changes in parasite morphology were observed by microscopic analysis. Moreover, using this new reformulation of the culture medium, the isolation of *B. bovis* from an asymptomatic carrier bovine was achieved (91).

Each component of the ins-tran-sel mixture performs specific functions; Insulin internalizes glucose to cells (94), transferrin collects and transports iron to erythrocytes for hemoglobin synthesis (95, 96), sodium selenite can function as an antioxidant and protector of the erythrocyte membrane (97, 98). In addition, A-DMEM/F12 contains hypoxanthine, which is vital for *in vitro* proliferation of protozoa (86, 99, 100) as some, including *B. bovis*, cannot synthesize hypoxanthine *de novo* (101). Another component of A-DMEM/F12 that facilitated the removal of bovine serum from the culture medium is ALBUMAX II, necessary for the proliferation of *Babesia* sp. (86, 89, 102). When the combination of medium A-DMEM/F12 with ins-trans-sel (2000, 1100, 1.34 mg/L) and Putrescine (Pu, 0.1012 mg/L) was used, maximal PPE of 17.26 and 14.8% were obtained for *B. bovis* and *B. bigemina*, respectively. With this culture medium combination, higher growth rates of parasite proliferation were shown (92, 93).

In these studies, it was demonstrated that there is an optimal concentration for Pu in the culture medium, by which the proliferation of *B. bovis* and *B. bigemina* can be best stimulated. It was also shown that Pu is required by both *Babesia* species to proliferate *in vitro* in a bovine serum free medium. This suggests that if parasites cannot synthesize Pu *de novo*, they may obtain it from the culture medium. All this indicates that precise concentrations of Pu should be used to stimulate the proliferation of *B. bovis* and *B. bigemina*.

**Figure 1:**
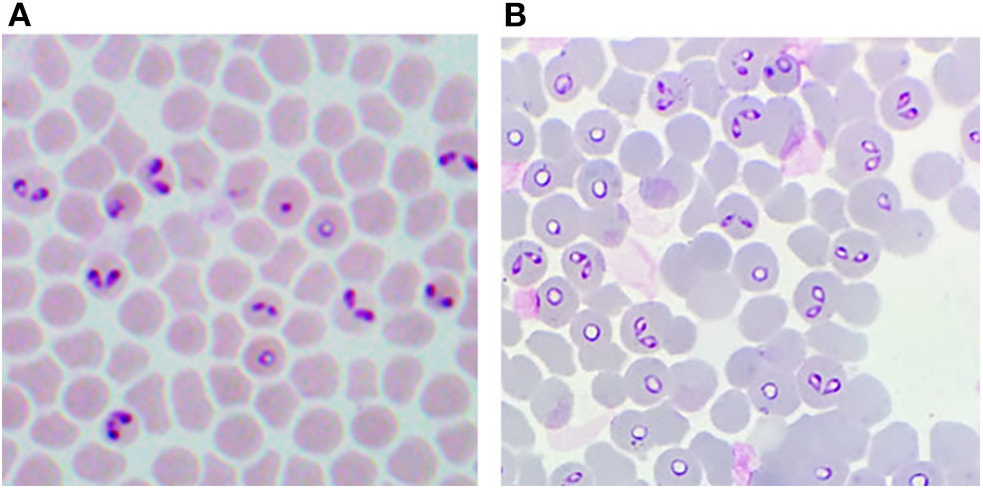
Giemsa-stained blood smear from *in vitro* cultured-derived *Babesia bovis*. **(A)** Conventional culture system. **(B)** Bioreactor culture system. 100X.

**Figure 2:**
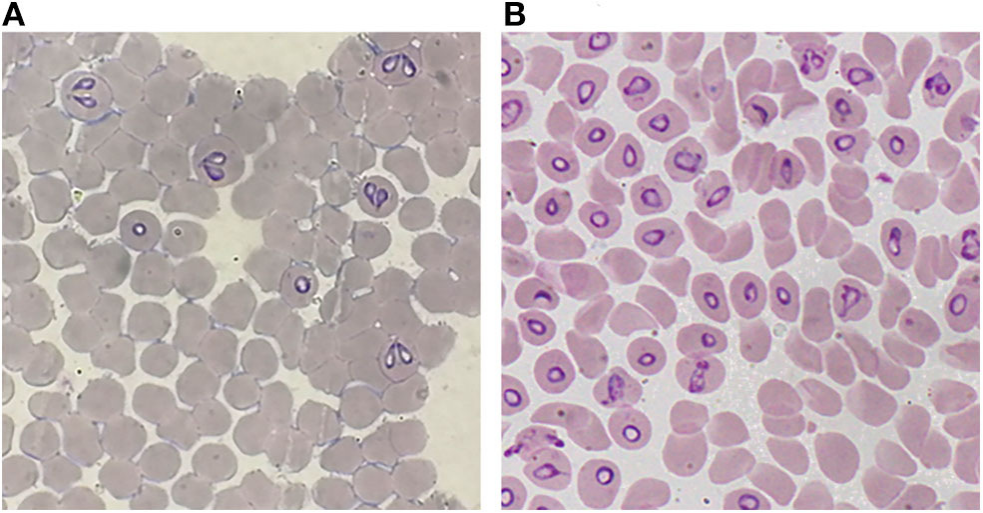
Giemsa-stained blood smear from *in vitro* cultured-derived *Babesia bigemina*, **(A)** Conventional culture system. **(B)** Bioreactor culture system.100X.

A decisive advance in the improvement of the efficiency for *in vitro* cultivation of *Babesia* spp. was the inclusion of a perfusion bioreactor, in addition to the achievements previously obtained. Thus, the A-DMEM / F12 medium, the mixture of ins-trans-sel, Pu, and particularly the lines of *B. bovis* and *B. bigemina* adapted to proliferate in a serum-free medium were combined. With these improvements, maximal PPE values of up to 29.7% for *B. bovis* (Figure 1) and 33.45% for *B. bigemina* (Figure 2) were reached. This was the first report of the cultivation of *Babesia* spp. with a perfusion bioreactor, demonstrating the production of *Babesia*-infected erythrocytes at high density (92, 93).

The freeze-thaw process employed in the *in vitro* culture system has been also modified to try to obtain a larger package of *B. bigemina*-infected erythrocytes and which could also be recovered in a shorter period once the *in vitro* culture were to be restarted from infected erythrocytes cryopreserved in liquid nitrogen (93). By modifying the freeze-thaw protocol in which the 20% PVP-40 culture medium was included in A-DMEM/F12 with 40% bovine serum, the *in vitro* culture system allowed parasite recovery in a short period. At 24 h post-thawing and seeding the cultures, parasites with typical morphology for the species were observed, which allowed establishing the continuity of the *in vitro* cultivation performing subcultures every 24 h, instead of every 72 h, as it has been reported previously using the standard methodology. Of the reported freezing systems, an efficiency of only 25% has been estimated for the recovery of *B. bovis* after the application of the freeze-thaw protocols (54). On the other hand, by testing the freezing protocol for *B. bigemina* it was reported that the recovery of parasites occurred at 48 h post-initiation of the culture, however no indication was made neither for the levels of infection, nor the continuous establishment of the parasites (59). Similarly, by following the standard methodology, PPE levels not exceeding 2% and only for short periods were later reported (61). The results obtained with this modification were probably due to the combination of the PVP-40 with the medium supplemented with serum and the activity of the serum proteins, which facilitated an adequate redox state of the erythrocyte membranes and the reduction of hemolysis (93).

## Importance and Immunoprophylactic Applications of *in vitro* Culture-Derived Parasites

Although there are different types of vaccines that have been tested against bovine babesiosis, so far the only procedure that has offered favorable results in terms of protection and safety has been the use of live attenuated vaccines in Australia (9, 27, 28). In other countries, such as Argentina, Brazil, Uruguay and Israel, this type of vaccine has also been produced and used (103).

The alternative to live vaccines other than splenectomized calf-derived biological material, has been the *in vitro* cultivation of *B. bovis* and *B. bigemina*. The development of the *in vitro* culture of *Babesia* spp. strains in a defined medium has been the basis for the initiation of a source of parasites for a variety of studies on the immunology of babesiosis. The various applications of cultivation include the selection of pure lines with different degrees of virulence (104) in order to obtain attenuated strains for vaccination (105–107). The use of live immunogens from attenuated strains derived from *in vitro* culture has been proposed as an alternative in the control of bovine babesiosis. In several studies, this type of immunogen applied to susceptible cattle has shown the induction of protection against an experimental heterologous challenge under controlled conditions (72, 105–107). Challenge of immunized cattle with the *R. microplus* tick has also been evaluated in cattle kept in natural conditions, by exposure of vaccinated animals to confrontations on vector-infested farms (34, 68).

## Experience on Live Attenuated Vaccines From *in vitro* Culture in Mexico

In Mexico, as previously described (4) bovine babesiosis is one of the most important diseases transmitted by arthropods to cattle in the tropics. Prevalence rates of up to 96% have been estimated in different livestock regions of the country by serological and molecular assays (35).

Among parasitic diseases, bovine babesiosis is one of the main obstacles to improve production, highlighted by its economic impact on the livestock activity. It is important to mention that out of the total cattle population in Mexico, estimated at about 30 million cattle heads, almost 75% are in areas infested with *R. microplus* ticks. Therefore, there is always the possibility of infection with *Babesia* spp.; as up to 89% of *Babesia* sp. antibodies prevalence rate has been reported in Mexico, thus considering to be a serious obstacle to the introduction of genetically improved cattle, for high bovine beef and milk production (1).

Since instrumentation of the *in vitro* cultivation system, which allowed for selection of various strains with different growth patterns *in vitro*, culture-derived parasites were then tested as live immunogens, for which the pathogenicity and immunogenicity of different *Babesia* spp. cell lines were evaluated. These early studies attempted to assess the reduced virulence of both, parasite lines and clones after being inoculated in susceptible animals (104, 108). Subsequent studies were carried out to determine the immunoprotective dose for the immunogen containing *B. bovis* parasites, in which different groups of cattle were inoculated at increasing doses, from 1 × 10^5^*to*1 × 10^9^ infected erythrocytes (IE). In these vaccine evaluation experiments, the variables registered have usually included: Determination of the packed cell volume (PCV) by the microhematocrit method; parasitemia expressed as percent of parasitized erythrocytes (PPE) and presence or absence of characteristic clinical signs of bovine babesiosis, basically fever, hemoglobinuria or mortality. Also, to establish the challenge dose with pathogenic parasites a virulent strain was obtained from a clinical babesiosis case, cryopreserved in liquid nitrogen and reactivated in a splenectomized calf. Then, increasing doses from 1 × 10^4^*to*1 × 10^8^ infected erythrocytes were assessed in terms of virulence. All these experiments included cattle inoculated with uninfected erythrocytes (UE) that were considered as a non-vaccinated control group. In this way, an inoculum containing 1 × 10^8^ *B. bovis*, or *B. bigemina*-infected erythrocytes was chosen as the challenge doses (33, 69). At challenge, none of the vaccinated animals showed clinical disease, whereas the control animals inoculated with UE showed severe clinical disease involving declines in PCV, fever (>40°C) and presence of parasites in peripheral blood smears. The most suitable vaccine dose selected was 1 × 10^7^ infected erythrocytes for both *Babesia* species (33, 69, 109).

Afterwards, and in order to prove cross-immunity between *B. bovis* and *B. bigemina*, cattle were inoculated with the monovalent *B. bigemina* or *B. bovis* vaccine and a third group with a combined immunogen containing both species; a control group was inoculated with UE. At challenge, protection assessed was 25, 50, and 100% for cattle immunized with *B. bigemina, B. bovis*, and *B. bigemina/B. bovis*, respectively. As for animals inoculated with UE, all were severely affected, one died, and the rest were treated to avoid unnecessary death. It was concluded that cross-protection is not good enough to induce solid immunity and, therefore it is necessary the application of the combined *B. bigemina*/*B. bovis* immunogen is necessary (110).

The bivalent immunogen containing fresh *B. bigemina* and *B. bovis* was evaluated under a controlled challenge. At 3 months post-vaccination (PV) animals showed slight decrease in the PCV with no changes in rectal temperature values and parasitemias were as low as 0.01 up to 0.06 PPE due to *B. bovis* and *B. bigemina*, respectively. In contrast, control group animals showed fever, decrease in PCV values of up to 29% and parasitemias as high as 0.5 for *B. bigemina* and 0.03 for *B. bovis*, respectively. Thus, adequate protection was induced by the combined live attenuated vaccine (109).

To make a continuous use of the attenuated live vaccine, another study in a two-phase experiment was conducted. First, cattle were injected with the combined vaccine and a control group with UE. Animals were lodged in a tick-free area for 2 months; then cattle were moved into a farm with 80% of seroprevalence to *Babesia* spp. As described for other evaluations, vaccinated cattle were moderately affected, but treatment was not required. In contrast, unvaccinated animals showed severe clinical signs, such as fever (41°C), decreasing PCV higher than 50%, and parasites were detected on the peripheral bloodstream by Giemsa stained smears (72). Another experiment in which the only difference was that the animals were vaccinated and kept in a ranch under hyperendemic conditions for *Babesia* spp., cattle were maintained in tick-restricted stables for 21 days after which animals were released into tick-infested pastures. Under these circumstances, protection was reduced to 70% in the vaccinated animals, although the entire control group showed very severe signs of the disease (34).

The attenuated vaccine evaluations were successful, both under controlled and field conditions. However, the handling and storage of fresh material showed serious limitations (7 days of half shelf life). Therefore, *B. bovis* and *B. bigemina* at different doses, from 1 × 10^7^ to 5 × 10^8^ IE were cryopreserved in 20% PVP in a liquid nitrogen tank. The inocula were injected in cattle, including as a positive control a group of cattle vaccinated with 1 × 10^7^ IE of fresh material. Vaccinated animals were then challenged in the field by tick exposure, finding a protection conferred of 90% in vaccinated cattle (35). Similar results were observed by applying the bivalent attenuated vaccine added with *Lactobacillus casei* in naive cattle (111).

An interesting evaluation of the attenuated vaccine in native cattle was carried out in an enzootically unstable farm for bovine babesiosis. Due to the excessive control of ticks, clinical and fatal cases had been described in this cattle farm. In that scenario, vaccination of cattle with frozen stabilates of culture-derived parasites helped restoring the enzootic stability. Therefore, the vaccine would be useful not only for prevention but also to control clinical outbreaks (112).

More recently, a live vaccine containing *B. bovis* and *B. bigemina*-infected erythrocytes cultured *in vitro* with a serum-free medium was assessed for protection conferred to naïve cattle, under natural tick-challenge in a high endemicity zone for *Babesia* spp. Vaccinated animals with Babesia-infected erythrocytes derived from both the serum-free culture system or the traditional bovine serum-containing culture system showed an excellent protection level, whereas the control unvaccinated animals were not protected and showed severe clinical signs, closely related to bovine babesiosis (113).

Studies on the improvement of the *in vitro* culture system of *B. bovis* and B. *bigemina* have demonstrated that it is possible to expand from a flask of 72 cm^2^ containing a PPE of 4% to a perfusion bioreactor, steadily increasing the maximal PPE up to 30% while harvesting material every 24 h. This has been achieved for both *B. bovis* and *B. bigemina* culture systems which implies obtaining a larger number of vaccine doses in a shorter period (92, 113).

Despite the development and continuous improvement for the *in vitro* culture methodology, currently there is not a commercially available vaccine in Mexico. However, farmers in enzootically unstable areas of bovine babesiosis have been utilizing the vaccine in validation programs to prevent babesiosis outbreaks, particularly on pure breed cattle that is translocated to tropical regions. In this sense, establishment of adequate farm production practices such as the vaccination strategy for disease prevention is essential.

## Experiences on Live Attenuated Vaccines From Other Countries

Ever since 1964, live attenuated vaccine strains of *B. bovis* and *B. bigemina* derived from splenectomized calves have been described in Australia (9). However, by using the vaccine produced by multiple passages in calves some failures have been described. Several studies have concluded that even though a high immunogenic response can be attained, the vaccine has a limited lifespan. Apparently, this is due in part to the presence of variant antigenic parasite populations in the field; thus, presenting the need for changing the vaccine strain every 3–5 years to prevent vaccine failures (114). It has also been described that around 27 million doses of vaccine had been utilized in Australia up until 1996 (115).

An attenuated Australian *B. bigemina* G strain was tested in South Africa, and although slightly affected upon vaccination, animals were protected after challenge with a virulent strain (116). In field trials, the immunity provided by the calf-derived attenuated vaccine is long lasting, observing only failures associated to immunogenicity of the strain, or lack of responsiveness for some animals. On the other hand, some disadvantages have been described for the use of calf-derived live vaccines including the development of acute disease, dissemination of other pathogens that are contaminants, as well as the sensitization of vaccinated animals leading to hemolytic anemias of newborn calves (28).

In Paraguay, pregnant heifers were vaccinated with the Australian vaccine strains. After a syringe challenge with local *B. bovis* parasites, vaccinated animals showed an effective immune response. However, the response to *B. bigemina* was not conclusive due to the apparent avirulent nature of the local strain (117).

Regarding *Babesia* vaccines using *in vitro* culture-derived parasites, Yunker et al. (105) described a virulent strain adapted to grow *in vitro* by using equine serum in the culture medium. It was demonstrated that after an undetermined number of serial passages *in vitro*, an attenuated *B. bovis* strain was attained which when inoculated in experimental animals, was able to induce solid immunity by challenging experimental cattle with a virulent strain 2 months after vaccination. Unvaccinated animals were severely affected, and nervous signs were observed.

In Argentina, an *in vitro* culture-derived *B. bigemina* vaccine, showed good protection in naïve cattle. Vaccinated animals were able to stand the virulent challenge without specific treatment. In fact, a vaccine which includes *B. bovis* and *B. bigemina* is currently commercially available (118).

Some drawbacks for the use of attenuated vaccines have been described that include the spread of adventitious pathogens, difficulties in dose standardization, parasite virulence reversion and quality control stringencies during the production and handling of the vaccines. However, vaccines containing *in vitro* culture-derived parasites are processed under more controlled conditions, and have a lower risk to acquire adventitious pathogens due to gamma radiation of the serum, and more recently by using bovine serum-free culture medium (31, 36, 91, 93). As for the handling of the vaccine inoculum, the usefulness of the vaccine as fresh or frozen material has been demonstrated (35, 119).

Despite many shortcomings the attenuated vaccines at the present time are the best strategy for bovine babesiosis prevention (31). *B. bovis* attenuated vaccines produced both *in vivo* or *in vitro*, are infective and can induce similar solid protection against virulent challenge (106, 107, 120, 121). Timms and Stewart (122) combined the stationary culture and suspension culture systems to scale *B. bovis* proliferation as a live vaccine. This procedure was recognized as a system providing for enough quantity and quality of parasites to be feasible for massive vaccination in the field.

Since the *B. bovis* genome was sequenced (101), transfection technologies have facilitated carrying out genetic studies in this parasite (123, 124). Furthermore, an attenuated vaccine with a genetically transfected strain would allow to easily discriminate among vaccinated and naturally infected animals in the field (125). By using transfection technologies combined with the *in vitro* culture system, there would probably be improvements in the production of genetically modified live vaccines (15, 126).

Currently, the research community is discussing on what type of methodology is needed for an effective vaccine against bovine babesiosis, beyond the methodology of *in vitro* cultivation of *Babesia* spp. One approach suggested is the use of a blood-stage vaccine to prevent clinical disease or to avoid transmission from tick to host. Another approach is a tick-stage vaccine to prevent parasite transmission between host and ticks (127).

## Future Perspectives

*In vitro* cultivation of *B. bovis* and *B. bigemina* provides for a wide number of applications: as a source of biological material for immunological studies, for the production of antigens for serological diagnostic techniques; moreover, it is an indispensable methodology for the development of subunit and recombinant vaccines, although currently one of the main benefits is the production of live attenuated vaccines. In spite of having been demonstrated its usefulness in terms of safety and protection, particularly in susceptible cattle introduced to hyperendemic regions of babesiosis in Mexico, the culture-derived live vaccine faces now a greater challenge, the parasite cultivation at large scale for mass production and distribution, due to the specific requirements for this type of vaccines.

## Author Contributions

JA and JF: conceptualization and writing, review and editing. JA, JF, and CR: formal analysis. JA and CR: writing original draft preparation. JF: project administration and funding acquisition. All authors contributed to the article and approved the submitted version.

## Conflict of Interest

The authors declare that the research was conducted in the absence of any commercial or financial relationships that could be construed as a potential conflict of interest.
